# Gentrification, measures of neighborhood change, and infant mortality in Michigan

**DOI:** 10.1038/s41372-026-02728-4

**Published:** 2026-06-01

**Authors:** Daria Murosko, Molly Passarella, Diana Montoya-Williams, Roshanak Mehdipanah, Scott Lorch

**Affiliations:** 1 Department of Pediatrics, Perelman School of Medicine, University of Pennsylvania, Philadelphia, PA USA; 2 Division of Neonatology, The Children’s Hospital of Philadelphia, Philadelphia, PA USA; 3 Children’s Hospital of Philadelphia PolicyLab, Philadelphia, PA USA; 4 Department of Health Behavior and Health Education, School of Public Health, University of Michigan, Ann Arbor, MI USA

**Keywords:** Epidemiology, Epidemiology, Paediatrics

## Abstract

**Objective:**

Determine the association of infant mortality (IM) and gentrification, a composite measure of rising neighborhood income, education, and housing costs.

**Study design:**

Retrospective cohort analysis of 672,432 infants born 2010–2019 across metropolitan Michigan. Multilevel multivariable regression models examined the association of gentrification and IM. Gentrification components were evaluated in separate models.

**Results:**

Gentrification was not associated with IM, but some of its components were. Living in a census tract with an above median rise in income or education attainment was associated with lower odds of IM, compared to below median change (Income: aOR=0.88; 95% CI: 0.82–0.95; Education: aOR=0.93; 95% CI: 0.86–0.997). Rent increases above the median were linked to higher IM among term infants and lower IM among infants <32 weeks’ gestation. The association of IM with rising home values varied by maternal insurance status.

**Conclusions:**

Indicators of increasing community affluence, often combined to define gentrification, have opposing relationships with IM.

## Introduction

Gentrification is a process of neighborhood change characterized by the influx of new, affluent residents into a historically disadvantaged community that results in the elevation of its socioeconomic position relative to a broader urban community [1]. Long-term residents in a changing neighborhood are often exposed to erosion of community connectivity, increased housing costs, and fear of displacement, contributing to heightened stress that may ultimately drive adverse health outcomes [2–4]. Infant health may be uniquely affected by gentrification. Financial hardship and familial stress, common in the newborn period, are already risk factors for sudden unexpected infant deaths and fatal child maltreatment [5, 6]. Neighborhood-level measures of housing instability and socioeconomic strain have been linked to infant mortality (IM), death prior to one year of age [7–9]. On the other hand, upward neighborhood mobility, defined by increasing neighborhood income, has been associated with reduced IM risk [10]. These patterns suggest that neighborhood change may simultaneously introduce both risks and resources, underscoring the need to directly examine the net impact of gentrification on IM.

To advance an understanding of gentrification and health, researchers must operationalize gentrification and identify where neighborhood shifts are occurring. Freeman’s widely used conceptual framework posits that gentrification only occurs in neighborhoods that are relatively deprived compared to the larger urban community [11]. Neighborhoods gentrify if they experience outsized improvements in socioeconomic measures over time. Common indicators include proportion of college-educated adults, median income, poverty rate, and age of housing stock, reflecting disparate views on which dimensions of neighborhood change are most salient [4, 12].

Heterogeneity in how gentrification is defined complicates efforts to characterize its relationship with health. Studies of preterm birth, the leading cause of IM, report associations in opposing directions [13–16]. While this inconsistency is partially attributable to differences in geographic regions and population case mix, each study used gentrification definitions that incorporated unique dimensions of neighborhood change. Prior work shows limited overlap in areas identified as gentrified across definitions [12]. In response, scholars have called for rigorous, context-specific conceptualizations of gentrification aligned with the specific research question [17].

One potential approach to understand drivers of gentrification is to disaggregate definitions of gentrification to examine single indicators of the neighborhood. Housing affordability and income mobility, for example, have been linked to infant health outcomes; however, it remains unclear whether changes in these components, often embedded within composite gentrification measures, are independently associated with IM or reflect part of the gentrification process. Accordingly, we examined the association between neighborhood gentrification and IM. Secondarily, we further disaggregated the composite gentrification definition to assess whether individual components of neighborhood change were independently associated with IM.

## Methods

### Study design and population

We conducted a retrospective cohort analysis using linked maternal-infant birth hospitalization, birth certificate, and death certificate data for all infants born in Michigan from 2010 to 2019. Eligible infants were born between 22 0/7 - 44 6/7 weeks' gestational age (GA). We excluded infants with missing or unlinked census tract data on birth certificates, as well as those with implausible birthweights (defined as >5 standard deviations from the mean for GA), to minimize data entry errors. Because gentrification has been most extensively analyzed in urban areas [3, 11], we excluded infants that did not reside (per the birth certificate) within 4 of the largest Metropolitan Statistical Areas in the state: Detroit (Wayne, Oakland and Macomb counties), Grand Rapids (Kent, Ionia, Ottawa, Montcalm and Barry counties), Lansing (Ingham, Eaton and Clinton counties), and Ann Arbor (Washtenaw county). The study was designed in accordance with the Strengthening the Reporting of Observational studies in Epidemiology guidelines, and the use of these de-identified data was considered non-human subjects research by the institutional review board [18].

### Exposures of interest

The primary exposure was residence in a neighborhood that gentrified between 2000 and 2010. This period was chosen to capture the long-term, possibly lagging effects of neighborhood change, and is aligned with other studies [3]. Like others, we defined neighborhood by census tract, which was determined by geocoded maternal address at time of delivery [19]. We obtained the 2000 Decennial Census and the 2010 5-year American Community Survey data from Social Explorer, which harmonizes census boundaries across different years, accounting for shifts in tract boundaries [20]. While many measures of gentrification exist, we selected the Ding definition that incorporates multiple measures, including educational status and housing costs, recognizing that economic change can occur over multiple dimensions [21]. Per Ding, neighborhoods were considered eligible for gentrification if they had greater than 50 residents and their 2000 median house income (MHI) was below the metropolitan median value. Neighborhoods with MHI greater than the 2000 median were designated as “Ineligible” to gentrify [21]. Eligible neighborhoods were designated as “Gentrified” if, between 2000 and 2010, the change in percent college completion was above the metro’s median, and if the 2000-2010 neighborhood percentage change in median self-reported home value or average rent was above the metro’s median [21]. Neighborhoods that did not meet both criteria did not gentrify.

Secondary exposures included the components of the gentrification index. We determined how much mean household income (MHI), educational attainment (% college completion), rent, and home values rose or fell in each neighborhood from 2000 to 2010, expressed as a relative percent change (difference between 2010 and 2000 values, divided by 2000 value). To enhance comparability between metro areas, neighborhoods were grouped into quartiles (Q) based on percent change for each component, with Q4 indicating the largest rise.

### Outcome of interest

We analyzed IM, defined as death prior to the first birthday.

### Covariate selection

We included demographic covariates previously associated with infant death or preterm birth: maternal age, education, smoking status, insurance, prenatal care receipt, as well as infant sex, gestational age, and birthweight (Table 1) [22–24]. We also included the metro area and year of birth. The experience of gentrification [13] and risk of IM [25] both vary by race and ethnicity, secondary to the relationship between structural racism and poverty [26] and their combined impact on birth and infant health outcomes that increase risk of IM. We included self-reported categories of race and ethnicity: Hispanic, Non-Hispanic Asian American/Pacific Islander (NHAAPI), Non-Hispanic Black (NHB), Non-Hispanic White (NHW), Multiple-Races, and Other Races and Ethnicities. To address missingness in maternal insurance payor, race and ethnicity, and education level, we created and incorporated missing category indicators [27].

**Table 1:** Gentrification exposure 2000–2010 and dyad and neighborhood characteristics, *n*(%).

Demographics	Did not gentrify (*n* = 202,927)	Gentrified (*n* = 141,360)	*p*-value^a^	Ineligible (*n* = 328,145)
Maternal Age, years (mean (SD))	27.16 (5.78)	27.15 (5.85)	0.53	29.96 (5.33)
Maternal Race/Ethnicity (*n* (%))			<0.001	
Hispanic	20,920 (10.3)	13,360 (9.5)		16,402 (5.0)
Multiple Races or Ethnicities	3379 (1.7)	2497 (1.8)		4452 (1.4)
NHAAPI	8007 (4.0)	4614 (3.3)		21,499 (6.6)
Non-Hispanic Black	77,565 (38.2)	54,483 (38.5)		30,851 (9.4)
Non-Hispanic White	90,156 (44.4)	64,569 (45.7)		251,093 (76.5)
Other Race or Ethnicity	2315 (1.1)	1490 (1.1)		2657 (0.8)
Missing	585 (0.3)	347 (0.3)		1191 (0.4)
Maternal Education (*n* (%))			<0.001	
No High School	7344 (3.6)	5286 (3.7)		2507 (0.8)
Some High School	30,532 (15.1)	22,818 (16.1)		13,579 (4.1)
High School or GED	63,131 (31.1)	44,236 (31.3)		51,342 (15.7)
At least some College	99,393 (49.0)	67,239 (47.6)		258,344 (78.7)
Missing	2527 (1.3)	1781 (1.3)		2373 (0.7)
Any smoking (*n* (%))	35,376 (17.4)	26,668 (18.9)	<0.001	40,332 (12.3)
Maternal Insurance (*n* (%))			<0.001	
Private	87,069 (42.9)	59,858 (42.3)		247,628 (75.5)
Government	1,13,510 (55.9)	80,031 (56.6)		75,506 (23.0)
Self-pay	1168 (0.6)	785 (0.6)		1994 (0.6)
Other	1180 (0.6)	685 (0.5)		3017 (0.9)
Missing	0 (0.0)	1 (0.0)		0 (0.0)
Adequacy of Prenatal Care (*n* (%))			0.077	
Inadequate	32,425 (16.0)	22,915 (16.2)		24,786 (7.6)
Intermediate	17,549 (8.7)	12,253 (8.7)		19,772 (6.0)
Adequate	65,191 (32.1)	44,890 (31.8)		1,23,548 (37.7)
Adequate-Plus	72,721 (35.8)	50,958 (36.1)		1,44,668 (44.1)
Missing	15,041 (7.4)	10,344 (7.3)		15,371 (4.7)
Infant Sex Male (n (%))	103,418 (51.0)	72,063 (51.0)	0.93	168,259 (51.3)
Metro Area (n (%))			<0.001	
Detroit	135,373 (66.7)	94,504 (66.9)		220,486 (67.2)
Ann Arbor	10,888 (5.4)	6582 (4.7)		18,048 (5.5)
Lansing	13,593 (6.7)	12,313 (8.7)		24,300 (7.4)
Grand Rapids	43,073 (21.2)	27,961 (19.8)		65,311 (19.9)
Gestational Age, weeks (median (IQR))	39 (38–40)	39 (38–40)	0.004	39 (38–40)
Birth Weight, kg (median (IQR))	3.25 (2.90–3.60)	3.25 (2.89–3.59)	0.002	3.37 (3.02–3.69)
Infant Death (*n* (%))	1355 (0.7)	918 (0.7)	0.51	1246 (0.4)
Components of gentrification, 2000				
Median household income (mean (SD))	48,868 (10,823)	46,195 (12,108)	<0.001	86,867 (22,742)
Percent bachelor’s degree (mean (SD))	16.78 (13.07)	12.71 (11.64)	<0.001	32.45 (16.27)
Average rent (mean (SD))	746 (159)	698 (134)	<0.001	996 (368)
Average home value (mean (SD))	117,581 (48,575)	107,018 (54,137)	<0.001	223,484 (88,161)
Percent change in components, from 2000 to 2010				
Median household income (mean (SD))	−28.4 (13.4)	−21.0 (0.15)	<0.001	−19.0 (11.4)
Percent bachelor’s degree (mean (SD))	−2.0 (32.5)	57.5 (47.8)	<0.001	18.3 (27.1)
Average rent (mean (SD))	4.7 (18.5)	14.2 (21.4)	<0.001	7.1 (30.2)
Average home value (mean (SD))	2.3 (25.6)	17.4 (43.3)	<0.001	6.7 (20.9)

*NHAAPI* Non-Hispanic Asian American/Pacific Islander.

^a^*p*-value for Did Not Gentrify vs Gentrified. Mean (SD) presented for maternal age. Median (interquartile range) presented for gestational age and birthweight.

### Statistical analyses

Descriptive statistics were examined by neighborhood gentrification (gentrified, non-gentrified, ineligible), using chi-squared tests for dichotomous variables and analysis of variance for continuous variables. To isolate the effects of change among deprived neighborhoods eligible to gentrify, we modeled IM in neighborhoods that gentrified compared to eligible neighborhoods that did not gentrify. A multilevel, mixed-effect logistic model included covariates for metro area, maternal demographics, and birth year (to account for temporal trends), with random effects for census tracts nested within metro areas (fixed effects) and with random intercepts for census tracts to account for patient clustering within census tracts.

We also examined descriptive statistics for each gentrification component (income, education, rent, and home value). We fit analogous mixed-effects logistic regression models to assess the relationship between IM and each gentrification component, modeled as above median change (Q3 and Q4) or below median change (Q1 and Q2), with a random effect for census tract to account for patient clustering. As one example, one set of models in these analyses looked for the association between IM and living in a neighborhood where the change in MHI was above the metro area median, compared to a neighborhood with an MHI change below the median.

The association between gentrification and health varies across metropolitan areas and may also differ by individual characteristics that are a proxy for exposure to structural inequities, influencing their exposure to and ability to adapt to neighborhood change. Accordingly, we assessed effect modification by testing interactions between gentrification (and its components) and metro area, maternal race and ethnicity, and maternal insurance payor, with *p-*values of <0.05 indicating significant interaction. Gestational age was also evaluated as a potential modifier, as susceptibility to certain causes of IM varies by gestational age. Finally, we repeated analyses for births occurring in 2010–2014 only, to determine if the association varied over time. *Code Availability:* Statistical analyses were performed using Stata version 18 (StataCorp, College Station, TX). Code can be made available by request to the authors.

## Results

Of 1,095,483 eligible births in Michigan between 2010 and 2019, 1,083,205 (98.9%) were linked to census indicators. Of these, 672,432 (62.1%) resided in the selected metro areas. 3519 died prior to their first birthday (0.52%). Overall, 48.8% lived in neighborhoods that were ineligible to gentrify; 30.2% lived in eligible but non-gentrified neighborhoods, and 21.0% lived in neighborhoods that gentrified between 2000 and 2010 (Table 1). Given the large sample size, there were statistically significant, but small, differences in demographics between infants from non-gentrified and gentrified neighborhoods. Neighborhood change indicators in 2000 were lower in eligible neighborhoods compared to ineligible neighborhoods. On average, all neighborhoods had declines in median household income between 2000 and 2010, but neighborhoods that gentrified had larger relative increases in educational attainment, average rent, and average home value, compared to both ineligible and non-gentrifying neighborhoods.

In both unadjusted and adjusted models, there was no association between the composite measure of gentrification and IM (Table 2). No significant interactions were found between gentrification status and gestational age, metro area, maternal race and ethnicity, or maternal insurance payor (Supplement 1). A sensitivity analysis limited to births between 2010 and 2014 also found no association between gentrification and IM (Supplement 1).

**Table 2:** Odds of infant mortality from 2010 to 2019 in neighborhoods that gentrified, compared to eligible neighborhoods that did not gentrify.

	Unadjusted OR (95%)	Adjusted Model aOR (95%)^a^
Gentrification	0.97 (0.88–1.08)	0.96 (0.88–1.04)
Metro Area		
Detroit		REFERENCE
Ann Arbor		0.69 (0.55–0.88)
Lansing		0.84 (0.70–1.01)
Grand Rapids		0.69 (0.61–0.79)
Maternal Race and Ethnicity		
Hispanic		0.88 (0.74–1.06)
Non-Hispanic Asian/Pacific Islander		0.89 (0.66–1.20)
Non-Hispanic Black		1.80 (1.62–1.99)
Non-Hispanic White		REFERENCE
Multiple Races		1.22 (0.86–1.71)
Other Race		1.24 (0.82–1.87)
Missing		1.54 (0.76–3.12)
Maternal education		
No high school		REFERENCE
Some high school		1.20 (0.92–1.55)
High school or diploma equivalent		1.01 (0.78–1.31)
At least some college		0.79 (0.61–1.02)
Missing		2.30 (1.65–3.22)
Maternal Age		
<18 years		1.19 (0.95–1.50)
18–35 years		REFERENCE
>35 years		1.13 (0.99–1.29)
Maternal Insurance		
Private		REFERENCE
Government		1.16 (1.05–1.27)
Self-Pay		1.53 (0.94–2.48)
Other		0.62 (0.26–1.49)
Any Smoking		1.33 (1.20–1.47)

*aOR* adjusted odds ratio, *95% CI* 95% confidence intervals.

^a^Adjusted model is also adjusted for year of birth (data not shown).

**Fig. 1:**
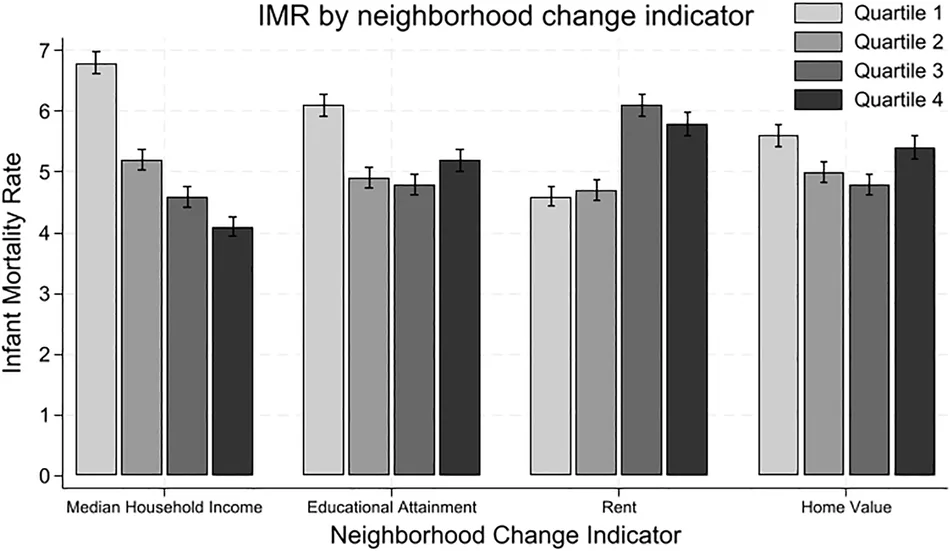
Neighborhood rates of infant deaths per 1000 live births, 2010–2019, by neighborhood change components. Neighborhood change is the relative census tract change in the indicator between 2000 and 2010. Quartile 1 represents the smallest, or least positive, rise in the indicator over the decade. Quartile 4 represents the greatest, or most positive, rise in the indicator. For Median Household Income, Educational Attainment, and Rent, *p* < 0.001. For Home Value, *p* = 0.06.

We next investigated the separate components of neighborhood change comprising the gentrification definition, stratifying neighborhoods by degree of change for each indicator (Table 3). In neighborhoods with the greatest rise in MHI, mothers were more likely to be older, Non-Hispanic White, have completed at least some college, and have private insurance. In contrast, in neighborhoods where rents rose the most, mothers were more likely to be younger, Non-Hispanic Black, have not completed high school, and have governmental insurance. IM rates varied with gentrification components. IM frequency fell from 6.8 to 4.1 per 1000 live births with rising neighborhood MHI (Fig. 1). Similarly, IM declined with rising neighborhood education. In contrast, IM rates increased from 4.6 to 6.1 per 1000 live births with rising neighborhood rent. No difference in IM was seen for rising neighborhood home values.

**Table 3:** Maternal and infant characteristics by gentrification component for births 2010–2019, stratified by neighborhoods of high vs low change,^a^ n(%).

	All neighborhoods	Median Household Income			College Attainment		
		Low change	High change	*p*-value	Low change	High change	*p*-value
Observations	672,432	357,298	315,134	—	330,412	342,020	—
Metro Area				< 0.001			< 0.001
Detroit	450,363 (67.0)	234,284 (65.6)	216,079 (68.6)		220,548 (66.8)	229,815 (67.2)	
Ann Arbor	35,518 (5.3)	20,924 (5.9)	14,594 (4.6)		16,196 (4.9)	19,322 (5.65)	
Lansing	50,206 (7.5)	24,387 (6.8)	25,819 (8.2)		22,602 (6.8)	27,604 (8.1)	
Grand Rapids	136,345 (20.3)	77,703 (21.7)	58,642 (18.6)		71,066 (21.5)	65,279 (19.1)	
Maternal Age				<0.001			<0.001
< 18 years	10,427 (1.6)	6843 (1.9)	3584 (1.1)		5315 (1.6)	5112 (1.5)	
18 – 35 years	557,848 (83.0)	302,222 (84.6)	255,626 (81.1)		273,778 (82.9)	284,070 (83.1)	
> 35 years	104,157 (15.5)			<0.001			<0.001
Maternal race and ethnicity				<0.001			<0.001
Hispanic	50,682 (7.5)	30,513 (8.5)	20,169 (6.4)		26,980 (8.2)	23,702 (6.9)	
Multiple Races	6462 (1.0)	3576 (1.0)	2886 (0.9)		3271 (1.0)	3191 (0.9)	
Non-Hispanic AAPI	34,120 (5.1)	17,206 (4.8)	16,914 (5.4)		18,683 (5.7)	15,437 (4.5)	
Non-Hispanic Black	162,899 (24.2)	113,131 (31.7)	49,768 (15.8)		87,720 (26.6)	75,179 (22.0)	
Non-Hispanic White	405,818 (60.4)	185,831 (52.0)	219,987 (69.8)		187,783 (56.8)	218,035 (63.8)	
Other race	10,328 (1.5)	5910 (1.7)	4418 (1.4)		4987 (1.5)	5341 (1.6)	
Missing	2123 (0.3)	1131 (0.3)	992 (0.3)		988 (0.3)	1135 (0.3)	
Maternal Education				<0.001			<0.001
No high school	15,137 (2.3)	9993 (2.8)	5144 (1.6)		7894 (2.4)	7243 (2.1)	
Some high school	66,929 (10.0)	43,823 (12.3)	23,106 (7.3)		33,541 (10.2)	33,388 (9.8)	
Completed high school^b^	158,709 (23.6)	97,496 (27.3)	61,213 (19.4)		78,293 (23.7)	80,416 (23.5)	
At least some college	424,976 (63.2)	201,671 (56.4)	223,305 (70.9)		207,271 (62.7)	217,705 (63.7)	
Missing	6681 (1.0)	4315 (1.2)	2366 (0.8)		3413 (1.0)	3268 (1.0)	
Smoking in pregnancy	102,376 (15.2)	58,379 (16.3)	43,997 (14.0)	<0.001	47,694 (14.4)	54,682 (16.0)	<0.001
Maternal Insurance				<0.001			<0.001
Private	394,555 (58.7)	182,782 (51.2)	211,773 (67.2)		190,975 (57.8)	203,580 (59.5)	
Medicaid	269,047 (40.0)	170,145 (47.6)	98,902 (31.4)		135,004 (40.9)	134,043 (39.2)	
Self-Pay	3947 (0.6)	2035 (0.6)	1912 (0.6)		1975 (0.6)	1972 (0.6)	
Other Insurance	4882 (0.7)	2335 (0.7)	2547 (0.8)		2458 (0.7)	2424 (0.7)	
Gestational Age Category				<0.001			0.38
> 37 weeks (term)	603,595 (89.8)	319,026 (89.3)	284,569 (90.3)		296,634 (89.8)	306,961 (89.8)	
32–36 weeks	57,673 (8.6)	31,825(8.9)	25,848 (8.2)		28,235 (8.6)	29,438 (8.6)	
< 32 weeks	11,164 (1.7)	6447 (1.8)	4717 (1.5)		5543 (1.7)	5621 (1.6)	
Birthweight,^c^ g	3306 (2950–3640)	3280 (2920–3615)	3340 (2990–3668)	<0.001	3295 (2948–3630)	3317 (2960–3651)	<0.001
Infant Death	3519 (0.5)	2154 (0.6)	1365 (0.4)	<0.001	1811 (0.6)	1708 (0.5)	0.006

	All neighborhoods	Median Rent			Median Home Value		
		Low change	High change	*p*-value	Low change	High change	*p*-value
Observations	672,432	351,577	320,855	—	349,493	322,939	—
Metro Area				< 0.001			< 0.001
Detroit	450,363 (67.0)	232,534 (66.1)	217,829 (67.9)		231,906 (66.4)	218,457 (67.7)	
Ann Arbor	35,518 (5.3)	18,838 (5.4)	16,680 (5.2)		18,107 (5.18)	17,411 (5.39)	
Lansing	50,206 (7.5)	25,955 (7.4)	24,251 (7.6)		25,108 (7.2)	25,098 (7.8)	
Grand Rapids	136,345 (20.3)	74,250 (21.1)	62,095 (19.4)		74,372 (21.3)	61,973 (19.2)	
Maternal Age				<0.001			<0.001
< 18 years	10,427 (1.6)	4014 (1.1)	6413 (2.0)		5581 (1.6)	4846 (1.5)	
18 – 35 years	557,848 (83.0)	290,938 (82.8)	266,910 (83.2)		294,476 (84.3)	263,372 (81.6)	
> 35 years	104 157 (15.5)			<0.001			<0.001
Maternal race and ethnicity				<0.001			<0.001
Hispanic	50,682 (7.5)	24,758 (7.0)	25,924 (8.1)		24,182 (6.9)	26,500 (8.2)	
Multiple Races	6462 (1.0)	3505 (1.0)	2957 (0.9)		4131 (1.2)	2331 (0.7)	
Non-Hispanic AAPI	34,120 (5.1)	23,186 (6.6)	10,934 (3.4)		17,562 (5.0)	16,558 (5.1)	
Non-Hispanic Black	162,899 (24.2)	64,066 (18.2)	98,833 (30.8)		93,483 (26.8)	69,416 (21.5)	
Non-Hispanic White	405,818 (60.4)	229,262 (65.2)	176,556 (55.0)		203,402 (58.2)	202,416 (62.7)	
Other race	10,328 (1.5)	5513 (1.6)	4815 (1.5)		5669 (1.6)	4659 (1.4)	
Missing	2123 (0.3)	1287 (0.4)	836 (0.3)		1064 (0.3)	1059 (0.3)	
Maternal Education				<0.001			<0.001
No high school	15,137 (2.3)	6269 (1.8)	8868 (2.8)		8230 (2.4)	6907 (2.1)	
Some high school	66,929 (10.0)	26,997 (7.7)	39,932 (12.5)		36,033 (10.3)	30,896 (9.6)	
Completed high school^b^	158,709 (23.6)	74,908 (21.3)	83,801 (26.1)		89,765 (25.7)	68,944 (21.4)	
At least some college	424,976 (63.2)	240,330 (68.4)	184,646 (57.6)		211 453 (60.5)	213,523 (66.1)	
Missing	6681 (1.0)	3073 (0.9)	3608 (1.1)		4012 (1.2)	2669 (0.8)	
Smoking in pregnancy	102,376 (15.2)	50,322 (14.3)	52,054 (16.2)	<0.001	54,427 (15.6)	47,949 (14.9)	<0.001
Maternal Insurance				<0.001			<0.001
Private	394,555 (58.7)	224,103 (63.7)	170,452 (53.1)		194,522 (55.7)	200,033 (61.9)	
Medicaid	269,047 (40.0)	122,326 (34.8)	146,721 (45.7)		150,431 (43.0)	118,616 (36.7)	
Self-Pay	3947 (0.6)	2142 (0.6)	1805 (0.6)		2188 (0.6)	1759 (0.5)	
Other Insurance	4882 (0.7)	3006 (0.9)	1876 (0.6)		2351 (0.7)	2531 (0.8)	
Gestational Age Category				<0.001			0.129
> 37 weeks (term)	603,595 (89.8)	317,302 (90.3)	286,293 (89.2)		313,658 (90.0)	289,937 (90.0)	
32–36 weeks	57,673 (8.6)	28,969 (8.2)	28,704 (9.0)		29,928 (8.6)	27,745 (8.6)	
< 32 weeks	11,164 (1.7)	5306 (1.5)	5858 (1.8)		5907 (1.7)	5257 (1.6)	
Birthweight,^c^ g	3306 (2950–3640)	3320 (2977–3657)	3289 (2928–3626)	<0.001	3290 (2948–3629)	3317 (2965–3656)	<0.001
Infant Death	3519 (0.5)	1617 (0.5)	1902 (0.6)	<0.001	1862 (0.5)	1657 (0.5)	0.26

*AAPI* Asian American/Pacific Islander.

^a^Neighborhoods are dichotomized by relative change in indicator between 2000 – 2010: Low change (below median change [quartiles 1–2]) or high change (greater than median change [quartiles 3–4]).

^b^Completed high school: high school diploma or degree equivalent.

^c^Birthweight: median (interquartile range).

In the multi-level mixed effects models of gentrification components, the patterns seen in the univariate analysis largely persisted (Table 4). Living in a neighborhood with an above median rise in MHI was associated with lower odds of IM in unadjusted models (OR 0.72, 95% CI: 0.66–0.78) and after adjustment (aOR 0.88, 95% CI: 0.82–0.95). Similarly, neighborhood rise in education was associated with lower odds of IM in unadjusted models (OR 0.91, 95% CI: 0.83–0.997), though this relationship was not statistically significant after adjustment. Conversely, living in a neighborhood with an above median rise in rent was associated with higher odds of IM in unadjusted models (OR 1.30, 95% CI: 1.19–1.41). This association was explained by differences in maternal demographic covariates in adjusted models (aOR 1.05, 95% CI: 0.98–1.13). There was no significant association between neighborhood rise in home value and IM.

**Table 4:** Adjusted associations of components of neighborhood change in metro Michigan with infant mortality from 2010 to 2019^a^.

	Median Household Income		College Attainment		Average Rent		Median Home Value	
	aOR (95% CI)^b^	Interaction term *p-*value^c^	aOR (95% CI)	Interaction term *p-*value	aOR (95% CI)	Interaction term *p-*value	aOR (95% CI)	Interaction term *p-*value
Unadjusted (*n* = 672 432)	**0.72 (0.66, 0.78)**		**0.91 (0.83, 0.99)**		**1.30 (1.19, 1.41)**		0.98 (0.90, 1.07)	
Total Adjusted^d^	**0.88 (0.82, 0.95)**		**0.93 (0.86, 0.997)**		1.05 (0.98, 1.13)		1.02 (0.95, 1.10)	
**Change in Indicator x Insurance**^e^		0.07		0.67		0.34		0.002
Private (*n* = 394,555)	0.83 (0.74, 0.92)		0.92 (0.82, 1.02)		1.01 (0.92, 1.13)		0.91 (0.82, 1.01)	
Medicaid (*n* = 269,047)	0.94 (0.85, 1.04)		0.94 (0.86, 1.04)		1.09 (0.98, 1.20)		**1.13 (1.03, 1.25)**	
**Change in Indicator x Metro**		0.26		0.70		0.10		0.60
Detroit (*n* = 450,363)	0.85 (0.78, 0.93)		0.92 (0.85, 1.00)		1.05 (0.97, 1.14)		1.04 (0.96, 1.13)	
Ann Arbor (*n* = 35,518)	0.89 (0.62, 1.29)		0.99 (0.70, 1.39)		1.60 (1.13, 2.27)		0.82 (0.58, 1.16)	
Lansing (*n* = 50,206)	1.05 (0.80, 1.37)		1.05 (0.80, 1.37)		1.14 (0.87, 1.50)		1.09 (0.83, 1.43)	
Grand Rapids (*n* = 136,345)	1.00 (0.83, 1.20)		1.00 (0.84, 1.20)		0.99 (0.82, 1.19)		1.02 (0.86, 1.23)	
**Change in Indicator x Maternal Race & Ethnicity**^e^		0.69		0.15		0.08		0.32
Non-Hispanic White (*n* = 405,818)	0.88 (0.80, 0.98)		0.96 (0.87, 1.06)		1.01 (0.91, 1.12)		1.01 (0.91, 1.12)	
Non-Hispanic Black (*n* = 162,899)	0.91 (0.81, 1.02)		0.88 (0.79, 0.98)		1.16 (1.04, 1.29)		1.11 (0.998, 1.23)	
Hispanic (*n* = 50,682)	0.94 (0.71, 1.24)		1.08 (0.82, 1.41)		0.90 (0.69, 1.18)		0.87 (0.67, 1.14)	
NHAAPI (*n* = 34,120)	0.71 (0.48, 1.06)		1.30 (0.88, 1.94)		1.44 (0.96, 2.14)		1.14 (0.77, 1.69)	
**Change in Indicator x gestational age**^f^		0.03		0.61		< 0.001		0.53
3 > 37 weeks (*n* = 603,595)	**0.80 (0.72, 0.90)**		0.90 (0.81, 1.01)		**1.22 (1.09, 1.37)**		1.06 (0.95, 1.18)	
32 < 37 weeks (*n* = 57,673)	0.85 (0.72, 1.01)		0.91 (0.78, 1.07)		1.07 (0.91, 1.27)		1.05 (0.89, 1.23)	
<32 weeks (*n* = 11,164)	0.98 (0.87, 1.10)		0.97 (0.87, 1.09)		0.90 (0.80, 1.00)		0.98 (0.88, 1.09)	

Bold denotes values that are statistically significant (confidence intervals not crossing 1).

*NHAAPI* non-Hispanic Asian American/Pacific-Islander.

^a^Neighborhood change components are the urban-specific relative increase of the following indicators between 2000 and 2010: (1) Median Household Income; (2) Proportion college attainment for population >25 years (College Attainment); (3) Average Rent; and (4) Median Home Value.

^b^Adjusted odds ratios (aORs) and 95% confidence intervals (95% CI) of infant mortality for mothers residing in neighborhoods with above the median rise in each indicator, compared to the below median change.

^c^Interaction terms were added to the model, separately, for gestational age and insurance. Reference groups were term infants (>37 weeks’ gestation) and private insurance. *p*values for the interaction terms are presented.

^d^All models are adjusted for metropolitan area, maternal race and ethnicity, maternal age (category), insurance payor, educational attainment, and smoking status.

^e^For ease of interpretation, insurance is dichotomized to private vs non-governmental insurance and race and ethnicity were restricted to the categories listed.

^f^For this model, gestational age was added as an interaction term, in addition to the covariables listed above.

There was effect modification by gestational age and maternal insurance status. An above median rise in neighborhood MHI and education was associated with decreased IM only among term infants ( > 37 weeks’ gestation) (Table 4). In contrast, an above median rise in neighborhood rent was associated with greater odds of IM among term infants, but with lower odds of IM among very preterm infants ( < 32 weeks’ gestation). There was no interaction by gestational age between the rise in home values and IM. However, the maternal insurance payor (governmental vs private insurance) modified the relationship between the rise in neighborhood home values and IM. Above median rise in home values was not associated with IM among infants whose mothers had private health insurance, yet among infants whose mothers had governmental insurance, an above median rise in home values was associated with IM (aOR 1.13, 95% CI: 1.03–1.25). There was no interaction between any neighborhood change component and metro area or maternal race and ethnicity. When restricting the sample to births from 2010 to 2014, effect estimates remained similar in magnitude, but confidence intervals widened due to a smaller sample size.

## Discussion

Infant mortality is a critical indicator of population-level maternal and infant health. The rate of IM in the US remains significantly greater than in other developed nations, which has been linked to neighborhood factors, including exposure to economic deprivation, social vulnerability, and residential segregation [9, 28–30]. While gentrification may improve some structural determinants of IM through increased economic investments that improve access to healthcare, healthy food or clean air, disruptions in community networks and increasing costs may increase housing instability, financial hardship, and stress in long-tenured, low-income pregnant residents and new parents [31, 32]. In this analysis of metro areas in Michigan, there was no association of gentrification, as defined by Ding, with IM. However, when components of the composite definition were analyzed, there were opposing relationships with IM: rising neighborhood incomes were associated with lower risk of IM, yet rising neighborhood housing costs were associated with higher risk of IM among term infants and those with lower socioeconomic status, reflecting the complex relationship between neighborhood change and health.

We assessed gentrification using a single, common definition, which may have implications for our analysis. Varying measures have yielded different associations with perinatal outcomes, underscoring inconsistencies with how gentrification is conceptualized [13–15]. Amid debate over which indicators are optimal for health research, we adopted a novel approach by disaggregating the composite definition of gentrification into its components, enabling a more nuanced evaluation of the neighborhood changes that may drive health effects.

Neighborhood-level rise in income was associated with lower odds of IM, aligned with prior work documenting this protective effect for individual-level rises in income and education [23, 33]. Similarly, analyses leveraging maternal residential mobility find that individual exposure to declining community-level affluence is a risk for IM and neonatal morbidity [34, 35]. One limitation of such analyses is that individuals who relocate may be different than those who stay [36]. Our cross-sectional, population-level analysis captures changing neighborhood-level exposures through serial measures of such factors in the same location over time, agnostic to mobility status. However, further longitudinal work is needed to determine if the protective effect is driven by reduced IM among long-tenured residents or by the in-migration of wealthier, well-educated individuals who, at baseline, have infants with lower risk of IM [23, 24].

In contrast, our results suggest that rising neighborhood housing costs (measured by rent and home values) have an opposing relationship with IM. Rising rent was associated with increased odds of IM among term infants, which constituted 89.8% of our cohort. The positive relationship between rising home values and IM persisted among dyads with governmental insurance, a proxy for low socioeconomic status. This divergence likely reflects fundamental differences in how housing cost pressures are experienced by renters versus homeowners [37]. Renters, who are disproportionately lower income and more sensitive to volatility in housing expenditures [37, 38], may be immediately impacted by rapid increases in rental prices. It is possible that by measuring neighborhood change over a decade, we did not capture the short-term effects of increasing rental costs; displacement may have already occurred [16]. In contrast, rising home values may signal sustained shifts in neighborhood socioeconomic composition, translating into increases in property taxes that may cost-burden low-income homeowners over time [37, 39].

Regardless of homeowner status, housing affordability is a fundamental determinant of family well-being, shaping not only financial stability but also health behaviors and outcomes. Eviction, a consequence of unaffordability, is highly correlated with adverse infant health [40]. Alternatively, families may make tradeoffs to maintain stable housing, such as reducing spending on nutritious food or forgoing medical appointments [41, 42]. As housing costs continue to skyrocket, these concerns may be exacerbated [43]. Unaffordable housing is associated with maternal morbidity, a critical risk factor for IM [44], and with increased psychosocial stress among families [45]. Yet these studies typically assess affordability at a single point in time, providing only a static view of a dynamic process. By linking longitudinal changes in housing costs with IM, our study advances this literature and underscores the importance of assessing neighborhood change as an exposure that evolves over time. Future analyses must continue to build on this understanding, investigating the exposures as continuous measures of rising costs and considering related indicators, such as housing cost burden.

We found that the relationship between neighborhood change indicators and IM varied by gestational age, which we hypothesize is due to disparate causes of mortality. For example, Sudden Unexpected Infant Death, the most common cause of IM in term infants, is highly correlated with structural determinants of health and maternal mental health [9]. In contrast, preterm infants are most likely to die due to prematurity complications before hospital discharge. IM among preterm infants may be less influenced by neighborhood context or its evolution over time, given the predominance of biologic and clinical risks associated with prematurity.

The strength of this analysis lies in the large and diverse sample size, particularly as we were able to merge state-wide individual data to granular indicators of neighborhood change. However, there are notable limitations. The scale at which to observe gentrification is frequently a matter of debate, in regard to both geographic scale and duration of analysis. Aligned with prior analysis, we analyzed metro areas consisting of multiple counties [46], recognizing that we identified gentrifying neighborhoods in suburban areas that likely have fewer adverse structural determinants of health, thus biasing our results to the null. Like other cross-sectional gentrification analyses, we were unable to determine which mothers may have been displaced from a changing neighborhood prior to delivery [15, 47]. Previous analyses have shown that the socioeconomic characteristics of displaced people are not substantially different from those of non-movers [21], but longitudinal analyses are needed to determine if there is significant misclassification of exposure in a subset of pregnant people and young children. Importantly, neighborhood change processes are inequitably distributed, reflecting historical and contemporary structural racism that disparately influences neighborhood access and housing choice. Further work must intentionally evaluate the complex processes through which gentrification relates to the entrenched racial inequities in infant mortality, leveraging spatial analyses to examine geographic patterning and differential impacts across communities.

However, this gap in understanding should not delay the design of programs that proactively protect infant health, especially as neonatologists are tasked with “following through” for their preterm patients by preparing families for transitions to home [48]. An understanding of shifting neighborhood-level structural factors is key to optimizing transition planning before hospital discharge. Although current best practices for infant care providers recommend screening for housing instability and referral to appropriate community resources, providers should be aware that existing federal housing assistance programs do not prioritize pregnant people or families with infants [49–51]. Our findings suggest that this omission represents a missed opportunity, because pregnancy and early infancy are periods of heightened susceptibility to the health effects of housing instability. Additionally, federal assistance programs supporting families with housing instability are inadequate [52]: policies must work “upstream” to prevent displacement through controlled economic development and zoning reform. Prioritizing pregnant individuals and families with infants within anti-displacement and housing policy could therefore serve as a dual investment: strengthening economic security while improving maternal and child health outcomes at a population level.

## Conclusion

In this retrospective analysis of metro areas in Michigan, one common composite measure of gentrification was not associated with IM. However, when we applied a novel approach of breaking the measure into its components, we found lower odds of IM in neighborhoods that had the highest rises in neighborhood income and education. Conversely, odds of IM were associated with rising home values among families with low SES and rising rent prices among term infants, together suggesting that increases in housing costs may be harmful to a subset of the population. Our results may be used to investigate the impact of affordable housing policies on infant health, particularly in vulnerable communities. Though it may be impossible and/or undesirable to slow the overall process of gentrification, policy experts may have more success in targeting specific interventions to preserve and expand affordable housing options in changing neighborhoods to mitigate any potential negative health sequelae associated with these changes.

## Supplementary information

Supplement 1

## Data Availability

The datasets generated during the current study are not publicly available because of the sensitive nature of the data, in accordance with the data use agreement and the local IRB. Requests regarding dataset access should be directed to the corresponding author. Code for the full dataset creation and analysis is available from the authors upon request.
