# Supplementary material for: Gentrification, measures of neighborhood change, and infant mortality in Michigan
Source: J Perinatol. 2026 Jun 1;46(10):1986–94. doi: 10.1038/s41372-026-02728-4 (PMC13680263; doi:10.1038/s41372-026-02728-4)
Supplement: Supplementary file 1 [file 41372_2026_2728_MOESM1_ESM.docx]

**Supplement 1. Adjusted associations of gentrification among eligible neighborhoods in metro Michigan with infant mortality from 2010 to 2019^1^**

|  | aOR (95% CI)^2^ | Interaction term *p-*value^3^ |
| --- | --- | --- |
| Change in Indicator x Insurance^4^ |  | 0.57 |
| Private (n= 394 555) | 0.93 (0.80, 1.08) |  |
| Medicaid (n= 269 047) | 0.98 (0.88, 1.08) |  |
| Change in Indicator x Metro |  | 0.79 |
| Detroit (n=450 363) | 0.93 (0.85, 1.03) |  |
| Ann Arbor (n=35 518) | 1.08 (0.68, 1.73) |  |
| Lansing (n=50 206) | 1.00 (0.71, 1.42) |  |
| Grand Rapids (n=136 345) | 1.04 (0.82, 1.33) |  |
| Change in Indicator x Maternal Race & Ethnicity^4^ |  | 0.11 |
| Non-Hispanic White (n=405 818) | 0.96 (0.82, 1.12) |  |
| Non-Hispanic Black (n=162 899) | 0.93 (0.83, 1.04) |  |
| Hispanic (n=50 682) | 1.04 (0.75, 1.44) |  |
| NHAAPI (n=34 120)^6^ | 1.92 (1.09, 3.40) |  |
| Change in Indicator x gestational age^6^ |  | 0.94 |
| >37 weeks (n=603 595) | 0.91 (0.79, 1.05) |  |
| 32 < 37 weeks (n= 57 673) | 0.94 (0.76, 1.16) |  |
| < 32 weeks (n= 11 164) | 0.94 (0.82, 1.08) |  |

^1^Eligible neighborhoods are those that were below metro median in Median Household Income in 2000, in accordance with the Ding definition. ^2^Adjusted odds ratios (aORs) and 95% confidence intervals (95% CI) of infant mortality for mothers residing in eligible neighborhoods that gentrified between 2000 and 2010, compared to eligible neighborhoods that did not gentrify. All models are adjusted for metropolitan area, maternal race and ethnicity, maternal age (category), insurance payor, educational attainment, and smoking status ^3^Interaction terms were added to the model, separately, for gestational age, insurance, Metro, and maternal race and ethnicity. *p*-values for the interaction terms are presented. ^4^For ease of interpretation, insurance is dichotomized to private vs non-governmental insurance and race and ethnicity was restricted to the categories listed. ^5^NHAAPI: Non-Hispanic Asian American/Pacific-Islander. ^6^For this model, gestational age was added as an interaction term, in addition to the the covariables listed above.
